# Social Adversity in Adolescence Increases the Physiological Vulnerability to Job Strain in Adulthood: A Prospective Population-Based Study

**DOI:** 10.1371/journal.pone.0035967

**Published:** 2012-04-25

**Authors:** Hugo Westerlund, Per E. Gustafsson, Töres Theorell, Urban Janlert, Anne Hammarström

**Affiliations:** 1 Stress Research Institute, Stockholm University, Stockholm, Sweden; 2 Department of Public Health and Clinical Medicine, Family Medicine, Umeå University, Umeå, Sweden; 3 Department of Public Health and Clinical Medicine, Epidemiology and Public Health, Umeå University, Umeå, Sweden; University of Granada, Spain

## Abstract

**Background:**

It has been argued that the association between job strain and health could be confounded by early life exposures, and studies have shown early adversity to increase individual vulnerability to later stress. We therefore investigated if early life exposure to adversity increases the individual's physiological vulnerability job strain in adulthood.

**Methodology/Principal Findings:**

In a population-based cohort (343 women and 330 men, 83% of the eligible participants), we examined the association between on the one hand exposure to adversity in adolescence, measured at age 16, and job strain measured at age 43, and on the other hand allostatic load at age 43. Adversity was operationalised as an index comprising residential mobility and crowding, parental loss, parental unemployment, and parental physical and mental illness (including substance abuse). Allostatic load summarised body fat, blood pressure, inflammatory markers, glucose, blood lipids, and cortisol regulation. There was an interaction between adversity in adolescence and job strain (B = 0.09, 95% CI 0.02 to 0.16 after adjustment for socioeconomic status), particularly psychological demands, indicating that job strain was associated with increased allostatic load only among participants with adversity in adolescence. Job strain was associated with lower allostatic load in men (β = −0.20, 95% CI −0.35 to −0.06).

**Conclusions/Significance:**

Exposure to adversity in adolescence was associated with increased levels of biological stress among those reporting job strain in mid-life, indicating increased vulnerability to environmental stressors.

## Introduction

Job strain, the combination of high demands and low control at work, has been shown to be associated with cardiovascular disease [1], [2], [3], depression [4], and a number of other health outcomes, especially among younger men [5]. However, it has been argued that the reported relationship between workplace factors and cardiovascular disease could be spurious and due to confounding by adversity in childhood [6]. For instance, a large Swedish study showed that the relationship between low job control and myocardial infarction risk could be statistically explained by adverse circumstances during childhood [7]. More recently, a study of the 1958 British Birth Cohort showed that workplace factors such as low job control and night work were associated with cardiovascular risk factors, but that 30–50% of this relationship was explained by early life exposures [8], and another study found socioeconomic differences in cardiovascular risk factors already among 10-year-old children in Britain [9]. However, an analysis of young Finns concluded that pre-employment factors did not confound the association between job strain and atherosclerosis two decades later [10], and a similar conclusion was drawn in a recent paper based on the Whitehall II study [11].

Unfavourable childhood conditions also seem to influence the way in which stress reactions are regulated, and epigenetic mechanisms [12], [13] as well as impact on telomere length [14] have been discussed. In addition, adverse childhood circumstances may increase the likelihood that a person will be exposed to bad working conditions – or at least perceive the conditions as bad - as an adult [15]. In line with this, a weak but statistically significant relationship between deficient emotional warmth in childhood and self-reported job strain was recently reported in a prospective Finnish study [16].

It has been argued that general stress mechanisms may be of importance for the relationship between job strain and myocardial infarction risk. For instance, one study found that job strain, particularly in combination with poor social support at work, was associated with increased risk of developing the metabolic syndrome [17], which is strongly related to long-lasting activation of stress mechanisms. Two components of the metabolic syndrome, high blood pressure [18], and total cortisol excretion during the waking hours [19], have also been discussed in this relationship.

The scientific divergence of opinions regarding the importance of job strain in the aetiology of cardiovascular disease warrants further studies of more complex relationships between childhood circumstances, job strain, and cardiovascular risk. Accordingly, the aim of the present study is, within the framework of a prospective population-based study, to examine if adversity in adolescence interacts with job strain in adult life to generate long-lasting stress responses. Adversity in adolescence was operationalised as closely as possible to ‘objective,’ mainly material, conditions in order to avoid reverse causality through subjective interpretations. The outcome variable that we have chosen to study is allostatic load, a composite measure of a general long-lasting activation of stress mechanisms [20] which may constitute a risk of future morbidity and mortality [21]. The hypothesis was that subjects with a history of adverse circumstances in childhood and adolescence would be more likely than others to show high allostatic load when exposed to job strain as adults.

## Methods

### Ethics

The longitudinal cohort study has been approved several times by Ethic Committees (the Ethics Committees of Uppsala University, Umeå University and Statistics Sweden as well as by the Regional Ethics Vetting Board in Umeå). Written consent has not been requested from these committees. The respondent is regarded as giving written consent when answering the questionnaire. The responders are always clearly informed that they can withdraw from the study whenever they wish, without giving any explanation.

### Population

The sample was based on the Northern Swedish Cohort, a 27-year prospective cohort study comprising all pupils in the ninth grade of the Swedish compulsory school living in Luleå in 1981, when the participants where 16 years of age (N = 1083; 506 girls and 577 boys) [22], [23]. Follow-up surveys were conducted in 1983, 1986, 1995 and 2008. In this report, data from the 1981 and 2008 surveys are presented. Of the original cohort, there were 1071 subjects still alive in 2008, of which 1010 (93.7%) agreed to participate. Of the 971 respondents in 2008, 158 were excluded since they stated that they were not gainfully employed at the time of the investigation, leaving 813 persons. Due to non-response on one or more key measures (see below), the effective sample size of the present report is 673 (at least 83% of the eligible participants and 72% of the original cohort minus those known not to be gainfully employed).

Participants completed a comprehensive questionnaire at all follow-ups. Although the composition of the questionnaire varied at different ages due to the age-specific relevance of some topics, the main areas covered in all versions included health, social and socioeconomic conditions, and school/working conditions. The majority of the items originated from the Swedish Survey of Living Conditions [24] and the Low-Income Study [25]. In 2008, a health examination was conducted, including blood pressure, anthropometrics, and the collection of blood samples after one night's fasting. The participants also performed saliva sampling four times during one weekday (at awakening, 15 minutes later, before lunch, and at bedtime) using Salivettes for the assessment of cortisol.

### Measurements

**Social adversity in adolescence** was operationalised as an index (range 0–6) counting the presence of the following exposures, based on the participants' responses at age 16: *Residential mobility:* The participants were asked how many times they had moved house in their lifetime. High residential mobility was defined as >2 relocations ( = 1), compared to 0–2 relocations ( = 0). *Residential crowding* was defined as the participant not having his/her own room at the time of the survey ( = 1). *Parental loss* was defined as ever having experienced either separation/divorce of parents, parents never living together, or death of either parent. *Parental unemployment* was defined as one or both parents being unemployed or granted a disability pension ( = 1) at the time of the survey (housewives were classified as employed). Values of >1 were recoded to 1. *Parental physical illness* was defined as one or both parents having a somatic illness ( = 1) at the time of the survey. Values of >1 were recoded to 1. *Parental mental illness* was defined as one or both parents having a mental health complaints or alcohol problems ( = 1) at the time of the survey. Values of >1 were recoded to 1. Since very few participants had four or more adversities in all (n = 13), 4–6 adversities were recoded to 3 in all analyses except the descriptive statistics.

**Job Strain** at age 43 was measured with a modified version of the Swedish Demand–Control Questionnaire (DCQ) [26], where ‘your work’ had been replaced with ‘your (main) occupation’ to allow also students, job seekers and others to respond. The questionnaire consists of 5 questions about Psychological Demands, 4 about Skill Discretion, and 2 about Decision Authority. All items have a four-point response option format, and the scores in each dimension were added together. To compute an overall job strain score, Psychological demands were divided into tertiles which were given the values of 0, 1, and 2. Decision Latitude (i.e. control) was defined as Decision Authority (excluding Skill Discretion) and the result was subsequently divided into tertiles, which were given the values 2, 1, and 0 from lowest to highest. The new scores for Psychological Demands and Decision Latitude were then added to form a composite job strain index ranging from 0 to 4 (Figure S1, left).

**Allostatic Load** at age 43 was operationalised as an index used previously and described in detail by our research group [27], based on the following 12 biological parameters: systolic and diastolic blood pressure, body mass index (BMI), waist circumference, fasting glucose, total cholesterol, HDL-C, triglycerides, Apo A1, Apo B, CRP, and cortisol area under the curve (AUC). Each parameter was divided into tertiles (coded 0, 1, 2), except cortisol AUC which was divided into sextiles and coded symmetrically (sextile 1 and 6 = 2, 2 and 5 = 1, 3 and 4 = 0). HDL-C cholesterol was coded inversely (2, 1, 0). To standardise for sex differences, recoding was done separately for women and men. Subsequently, mean scores of the parameters were calculated within six physiological systems: cardiovascular regulation, body fat deposition, lipid metabolism, glucose metabolism, inflammation, and neuroendocrine regulation. Pharmacological treatment was coded as 2 on the affected physiological system categories. Since most drop-out on biological parameters was due to failure to complete saliva cortisol sampling (n = 130), those without valid cortisol data were assigned the mean value 1 on the neuroendocrine category. Finally, the allostatic contributions of the physiological systems were summed up into an index (range of 0–12), yielding the final measure of allostatic load (Figure S1, right).

**Covariates** included in the study were sex, and socioeconomic status (SES) derived from the occupations stated by the respondents at age 43 and coded according to the socioeconomic classification system of Statistics Sweden [28]. In addition, we used the question ‘What is your current labour market position?’ and the response option ‘working gainfully’ to assess who were economically active at the time of the survey.

### Statistical Analysis

Bivariate Pearson correlation coefficients were calculated for all pairs of variables included in the study. In order to examine the associations between adversity in adolescence, job strain, and allostatic load, we used hierarchical linear regression based on Z-transformed variables in order to obtain 95% confidence intervals for standardised beta coefficients (β). Adversity in adolescence and job strain were added in Model 1. The interaction term adversity*job strain was entered in Model 2, and in Model 3 SES was added. Since the outcome had been standardised separately for men and women, sex was not included in the main analyses (after confirmation that there was no association between sex and allostatic load). The regression coefficients obtained for multiplicative interactions and reported with 95% confidence intervals, however, cannot be interpreted as standardised values, which is indicated by the Roman letter B. To study the possible impact of partial non-response, we generated five new datasets with multiple imputation and repeated the main analysis on these, and then compared the pooled results from the five imputed datasets with the results from the corresponding analysis in the original data. In order to illustrate the interaction between adversity in adolescence and job strain, we plotted the mean values of allostatic load for each level of job strain among those with a score of 0–1 and 2–5 on adversity in adolescence, respectively. All analyses were done in SPSS (PASW Statistics) for Windows, version 18.0.0, and all significance tests are based on two-sided tests.

## Results

There were 343 women and 330 men in the analytic sample; 35% of them were manual workers, and 77% had experienced less than 2 out of 6 possible adversities in adolescence (Table 1).

**Table 1:** Descriptive statistics for the participants with complete data on the main variables in the study.

	Women (N = 343)	Men (N = 330)	Total sample (N = 673)
	N (%)	N (%)	N (%)
**SES**			
professionals and higher managers	54 (16)	60 (18)	114 (17)
technical, lower management	125 (36)	102 (31)	227 (34)
non-manual	65 (19)	31 (9)	96 (14)
skilled manual	40 (12)	69 (21)	109 (16)
unskilled	59 (17)	68 (21)	127 (19)
**No. of adversities in adolescence**			
0	148 (43)	152 (46)	300 (45)
1	111 (32)	104 (32)	215 (32)
2	55 (16)	52 (16)	107 (16)
3	22 (6)	18 (6)	40 (6)
4	6 (2)	4 (1)	10 (2)
5	1 (0)	0 (0)	1 (0)

As can be seen in Table 2, adversity in adolescence was negatively associated with SES and positively associated with job strain and allostatic load at age 43 in women but not in men. The dimensions within the Job Strain Model and SES were related as expected.

**Table 2:** Bivariate correlations between the main variables in women (above diagonal, n = 334) and men (below diagonal, n = 325).

	1. AA	2. JS	3. PD	4. SD	5. DA	6. SES	7. AL
1. Adversity in Adolescence (AA)	1	**0.13** *	0.05	−0.09	−0.11	**−0.18** **	**0.12** *
2. Job Strain (JS)	0.05	1	**0.68** **	**−0.15** **	**−0.65** **	−0.05	0.06
3. Psychological Demands (PD)	0.09	**0.64** **	1	0.09	−0.07	0.03	0.04
4. Skill Discretion (SD)	−0.02	**−0.16** **	**0.24** **	1	**0.41** **	**0.37** **	**−0.11** *
5. Decision Authority (DA)	0.06	**−0.60** **	0.07	**0.49** **	1	**0.14** *	−0.10
6. Socio-economic status (SES)	−0.09	−0.06	**0.14** **	**0.37** **	**0.30** **	1	**−0.18** **
7. Allostatic Load (AL)	0.08	**−0.13** *	−0.07	**−0.11** *	0.07	**−0.18** **	1

* p<0.05;

** p<0.01.

**Figure 1:**
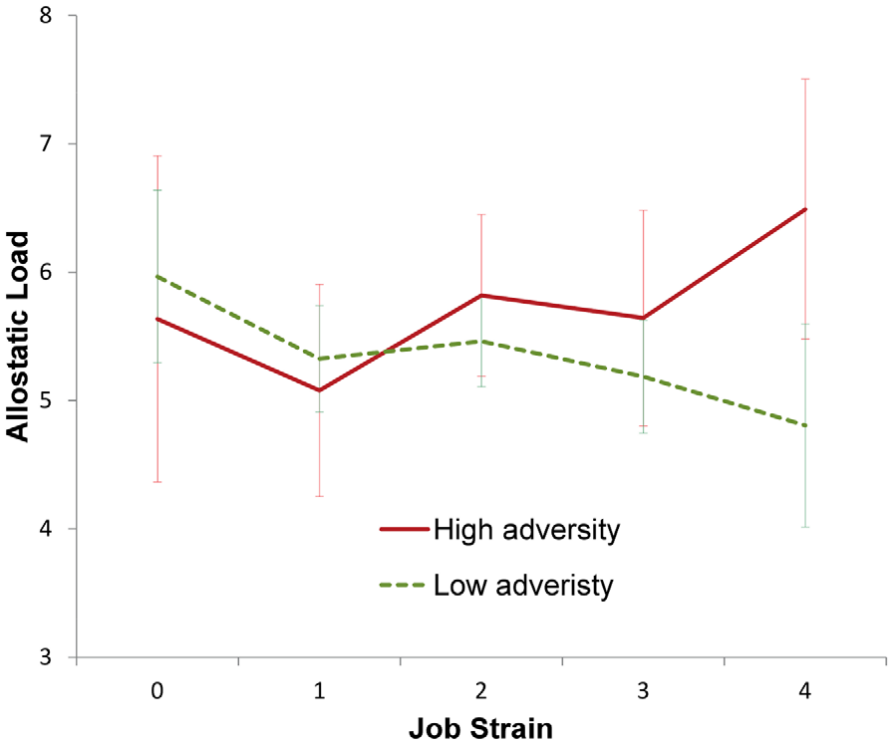
Interaction between adversity in adolescence and job strain in relation to allostatic load. Error bars indicate 95% confidence intervals.

Table 3 shows that there was a significant association between the amount of adversity in adolescence and allostatic load at age 43 in the total sample also after adjustment for job strain. Further adjustment for SES rendered the relationship non-significant. Job strain was not associated with allostatic load. However, there was a significant interaction between allostatic load and job strain, indicating that the former increases the physiological vulnerability to job strain. Fig. 1 indicates that the effect of high job strain differs between those who had been exposed to many adversities in adolescence and those who had not. We repeated the analyses on data where multiple imputation had been performed, and found that the interaction term between adversity in adolescence and job strain remained significant in the pooled analyses (p = 0.044); but in contrast to the main analysis job strain was found to be significantly negatively associated with allostatic load, whereas the main effect of adversity in adolescence did not remain significant after adjustment for the interaction term.

**Table 3:** Linear regression relating allostatic load to current job strain, including sub-components, and adversity in adolescence.

	M0: Univariate	M1: Mutually adjusted	M2: M1+interaction	M3: M2+SES
	B (95% CI)	B (95% CI)	B (95% CI)	B (95% CI)
**Job Strain**				
Job strain	−0.03 (−0.11 to 0.05)	−0.04 (−0.12 to 0.04)	−0.05 (−0.12 to 0.03)	−0.05 (−0.13 to 0.02)
Adversity in Adolescence	**0.10** (0.03 to 0.18)	**0.10** (0.03 to 0.18)	**0.09** (0.02 to 0.17)	0.07 (−0.00 to 0.15)
Interaction adversity*job strain			**0.09** (0.02 to 0.17)	**0.09** (0.02 to 0.16)
SES at age 43				**−0.17** (−0.25 to −0.10)
Adjusted R^2^		0.009	0.016	0.044
**Psychological Demands**				
Psychological Demands	−0.01 (−0.09 to 0.06)	−0.02 (−0.10 to 0.05)	−0.03 (−0.10 to 0.05)	−0.01 (−0.09 to 0.06)
Adversity in Adolescence	**0.10** (0.03 to 0.18)	**0.10** (0.03 to 0.18)	**0.10** (0.02 to 0.17)	0.07 (−0.00 to 0.15)
Interaction adversity*demands			**0.09** (0.01 to 0.16)	**0.08** (0.00 to 0.15)
SES at age 43				**−0.17** (−0.24 to −0.09)
Adjusted R^2^		0.008	0.014	0.040
**Decision Authority**				
Decision Authority	−0.01 (−0.09 to 0.06)	−0.01 (−0.09 to 0.06)	−0.01 (−0.09 to 0.06)	0.03 (−0.05 to 0.10)
Adversity in Adolescence	**0.10** (0.03 to 0.18)	**0.10** (0.02 to 0.18)	**0.10** (0.02 to 0.18)	**0.08** (0.00 to 0.15)
Interaction adversity*decision authority			−0.01 (−0.08 to 0.07)	−0.02 (−0.09 to 0.06)
SES at age 43				**−0.11** (−0.17 to −0.05)
Adjusted R^2^		0.007	0.006	0.034
**Skill Discretion**				
Skill Discretion	**−0.11** (−0.18 to −0.03)	**−0.10** (−0.18 to −0.02)	**−0.10** (−0.18 to −0.02)	−0.04 (−0.12 to 0.04)
Adversity in Adolescence	**0.10** (0.03 to 0.18)	**0.09** (0.02 to 0.17)	**0.09** (0.02 to 0.17)	**0.08** (0.00 to 0.15)
Interaction adversity*skill discretion			0.01 (−0.07 to 0.08)	0.01 (−0.07 to 0.08)
SES at age 43				**−0.11** (−0.17 to −0.05)
Adjusted R^2^		0.017	0.016	0.035

Among the three sub-dimensions of the Demand-Control Model (Table 3), Skill Discretion was negatively associated with allostatic load, also after adjustment for adversity in adolescence. Further adjustment for SES, however, rendered this association non-significant.

The interaction between adversity in adolescence and job strain found for the overall index was present for one subscale only, namely Psychological Demands. For the other subscales, the interaction was clearly non-significant and very close to 0, indicating that the overall interaction was almost exclusively due to an interaction between adversity in adolescence and Psychological Demands.

In order to examine whether the results were applicable to both women and men, we also stratified the analyses by sex (Table 4), which showed that the association between adversity in adolescence and allostatic load was significant in women but not in men and became non-significant also in women after inclusion of the interaction between adversity in adolescence and job strain. Job strain was associated with lower allostatic load in men but not in women. The interaction between adversity in adolescence and job strain did not reach significance in the analyses split by sex, but similar estimates of regression coefficients in the final models, and p-values of 0.085 (t = 1.760, df = 325) and 0.146 (t = 1.459, df = 338), indicate that this is due to lack of power.

**Table 4:** Linear regression relating allostatic load to current job strain and adversity in adolescence, split by sex.

	M0: Univariate		M1: Mutually adjusted		M2: M1+interaction		M3: M2+SES	
	B	95% CI	B	95% CI	B	95% CI	B	95% CI
**Women**								
Job Strain	0.06	(−0.04 to 0.17)	0.05	(−0.06 to 0.16)	0.04	(−0.07 to 0.15)	0.04	(−0.07 to 0.14)
Adversity in Adolescence	**0.12**	(0.02 to 0.23)	**0.12**	(0.01 to 0.22)	0.10	(0.00 to 0.21)	0.08	(−0.03 to 0.18)
Interaction adversity*job strain					0.09	(−0.02 to 0.20)	0.08	(−0.03 to 0.19)
SES at age 43							**−0.16**	(−0.26 to −0.05)
Adjusted R^2^				0.012		0.016		0.037
**Men**								
Job Strain	**−0.18**	(−0.32 to −0.03)	**−0.18**	(−0.33 to −0.04)	**−0.19**	(−0.33 to −0.04)	**−0.20**	(−0.35 to −0.06)
Adversity in Adolescence	0.08	(−0.03 to 0.18)	0.08	(−0.02 to 0.19)	0.08	(−0.03 to 0.19)	0.06	(−0.04 to 0.17)
Interaction adversity*job strain					0.11	(−0.03 to 0.25)	0.12	(−0.02 to 0.26)
SES at age 43							**−0.19**	(−0.30 to −0.08)
Adjusted R^2^				0.018		0.023		0.055

To examine whether women and men differed in the estimates, the main analysis was rerun with sex added as a predictor and with all possible two- and three-way interaction terms including sex. The results showed that women and men did not differ significantly in allostatic load (t = 0.700; p = 0.484). However, there was an interaction between sex and job strain (t = −2.383; p = 0.017), indicating that job strain was associated with allostatic load in men but not in women, as shown in Table 4.

## Discussion

In a prospective population-based cohort, exposure to adverse social conditions in adolescence, measured at age 16, was associated with increased vulnerability to job strain at age 43, reflected in a stronger association between job strain and allostatic load. This was largely explained by an interaction between adversity in adolescence and psychological demands in adulthood, indicating that the ability to cope psychologically, behaviourally or physiologically with the demands in working life may be affected by exposures in early life.

A major strength of this prospective study is that it is based on a stable cohort with very low attrition over the 27 years of follow-up. The cohort is population-based and has in various comparisons been found to be representative of the Swedish population [22]. Another strength is that three different types of data are used, decreasing the risk of common method variance: adversity in adolescence, although self-reported, was based on questions about objective facts, e.g. the number of times the respondent had moved house and whether a parent is physically ill. Job strain, measured 27 years later, is a self-assessment of the work environment worded to focus on the environment rather than individual perceptions [29]. Allostatic load was operationalised as an index of biological parameters which had first been divided into tertiles, thus emphasising variation within the asymptomatic spectrum, making reverse causality unlikely. Our study also has some limitations. Despite very high overall response rate, a relatively large number of participants did not complete the medical screenings at age 43, which could lead to biased results. Attrition analyses of adversity in adolescence, job strain and allostatic load revealed that job strain was 0.26 standard deviations lower among those who responded to the questionnaire at age 43 but did not have complete data (n = 172) compared with those who had complete data, including physiological screening, (p = 0.002), but there was no difference in adversity in adolescence between those who had complete data at age 43 and those who dropped out before the last follow-up. In addition, earlier analyses have shown that those with incomplete data were largely similar in both history of adversity and SES across the life course [30], in blood pressure and BMI in adolescence, as well as in adult health behaviours [31]. Importantly, this suggests that there was no systematic attrition of those with most unfavourable social circumstances in adolescence or adulthood, but rather that those with more favourable work circumstances in adulthood tended not to complete the physiological screening. Although the estimated main effect of job strain could potentially be affected, it seems unlikely that the key estimate – the interaction effect between job strain and adversity – would be biased as a consequence of this participation bias. Among those who took part in the physiological screening, allostatic load was 0.56–0.61 standard deviations higher among those who had missing data on cortisol, both when cortisol had been imputed (p<0.001) and not imputed (p<.001). This supports our decision to include also those not completing the saliva collection in the analyses, and indicates that their inclusion would not be expected to impact on the estimates. Furthermore, the multiple imputation analysis supported the results on the actual data, which gives further strength to the inferences.

By summarising several physiological systems in an allostatic load index, we decrease the risk that poor precision in one measurement substantially influences the results. However, although the operationalisation of allostatic load is based on the theory and literature on allostatic load, the construction of the index was constrained by limitations in our data, such as uneven number of markers from the different physiological systems. In addition, some measures, notably cortisol, is strongly influenced by day-to-day variations in environmental exposures, leading to imprecision in the measurement. Due to economical and practical constraints particularly relevant for epidemiological studies [32], a one-day saliva sampling protocol was used. Although sampling over at least two days may be optimal for precise measurement of the stable portion of the circadian rhythm [33] the relatively large sample size would be expected to partially counter the lack of precision resulting from the simple sampling protocol. Nevertheless, of the biological measures in the present study, it is important to note that the cortisol AUC is definitely the most difficult one to measure, and due to the single-day sampling we have no means to assess its reliability. The precision of the allostatic load index is therefore a limitation, which it has in common with other studies measuring allostatic load at one time point. Nevertheless, even allostatic load measured at one time point has been shown to predict subsequent morbidity and mortality [21]. Moreover, since we cannot see any reason why there would be systematic bias in this imprecision in relation to the studied exposures, the likely consequence is that the associations reported in this paper are lower than they would have been if the outcome had been measured with higher precision.

We cannot rule out that the association between adversity in adolescence and vulnerability in later life could be due to confounding. Childhood circumstances could be related to genetic factors that also interact with job strain in generating an elevated risk. It has for instance been shown that job strain was associated with increased thickness of the carotid artery wall among the 40% of middle-aged men who had the T/T genotype of Neuregulin-1, implicated in the regulation of stress responses, but not among men with other allele combinations [34].

There is, however, increasing evidence that inadequate social circumstances early in life can induce physiological vulnerability to insults later in life, e.g. by enduring adaptations of neuroendocrine and immunological regulation [35], [36]. As demonstrated in animal models, DNA methylation of key genes might explain such lasting physiological effects of a poor early environment [37]. One explanation for our findings could thus be an originally adaptive biological programming which becomes maladaptive in adulthood. However, there are also possible psychological and behavioural pathways between adversity in adolescence and increased vulnerability to stress, for instance through sustained arousal due to inadequate coping in individuals who have learned a pattern of ‘helplessness’ or ‘hopelessness’ [38] in an environment characterised by objective adversity. In humans, stress sensitisation related to history of childhood adversity has indeed been shown in relation to mental disorders, mainly depression [39].

Increased vulnerability to stress could also explain the main effect of adversity in adolescence on allostatic load in adulthood, as it could result in a cumulative burden of maladaptive stress reactions. An alternative, or indeed complementary, explanation for the main effect could be that those who are exposed to adversity early in life are also at greater risk of adverse exposures in adulthood [15], a relationship which may or may not be causal in the individual case.

As we have reported recently [30], there was also a direct effect of adversity in adolescence on allostatic load among women, indicating that adverse social circumstances can have long-term biological effects. This is in agreement with observations in cross-sectional studies on adults [40], [41] and children [42], [43]. Our findings are also in accordance with studies showing enduring effects of early adversity on health [44], [45], [46], as allostatic load may represent a pre-morbid state resulting from deleterious effects of poor social conditions in childhood.

Our finding of a negative relationship between job strain and allostatic load in men could be a spurious finding, considering both the null result in the whole cohort and the fact that previous cross-sectional studies on the relationship between job strain and biological risk factors for CHD have shown inconsistent results, with mostly null findings, plus some positive as well as negative findings. For instance, even though a study of employees in Stockholm (the WOLF study) showed that subjects with job strain tended to have lower HDL cholesterol, and female participants with job strain tended to have a higher prevalence of hypertension, these findings were weak and not consistent across age groups [47]. Findings have been slightly more consistent regarding immune parameters such as plasma fibrinogen than for cardiovascular risk factors in general but there are still inconsistencies, for instance with regard to gender specific findings [48]. The literature thus suggests that most of the relatively consistent relationship between job strain and incidence of cardiovascular disease [1], [2], [3] may have to be explained by other mediators than conventional biological risk factors. Another reason for these inconsistencies could be that most studies, like the present one, have measured job strain only once, which may be a poor indicator of accumulated exposure [49]. Thus, a study of British civil servants showed that those participants who had stated a combination of job strain and poor social support at 3 or 4 out of 4 measurement occasion over a 14-year period were twice as likely as the others to have metabolic syndrome (according to international standard, a measure similar to allostatic load) even after adjustment for risk factors such as poor diet and smoking [17]. Another study showed a relationship between cumulative exposure to job strain and systolic blood pressure in men [50]. Lower allostatic load among those with high job strain could also be a result of stronger health selection in the more demanding jobs.

A previous study examining allostatic load and work conditions found that only job demands were related to allostatic load [51]. These results are compatible with our finding, where the interaction effect seemed to be related specifically to the demand component of job strain.

In the present paper, we chose to use both the combination of high psychological demands and low job control, as well as the individual subscales of the Demand-Control Model. According to the theory behind the model [52], high demands are more difficult to handle when the possibility for the individual to make decisions are small. In prospective studies the job strain combination has been the most successful predictor of myocardial infarction, although during later years psychological demands have gained importance over both decision latitude and the job strain combination in predictions [3]. Current literature therefore provides rationales for studying both the job strain combination and the two main components in the demand control model.

One of the most important methodological differences between studies that have been published on job strain and health outcomes relates to the definition of job control, the denominator in the job strain formulation. It has been pointed out that during recent years in the post-industrial society skill discretion has changed its meaning. While a high skill discretion may still be regarded for many employees as a beneficial factor, it is increasingly regarded as a psychological demand factor for others, which may result in biased estimates of job strain [53]. In line with this, the study which has published the most authoritative findings, the Whitehall II study [11], [17], [49], has consistently used decision authority as equal to job control (excluding the skill discretion component). We therefore chose not to include skill discretion in our index of job strain. However, a sensitivity analysis using the original job strain formulation, with skill discretion included, yielded a non-significant interaction term between job strain and adversity in adolescence (B = 0.06, 95% CI −0.01 to 0.14, in Model 3) and no main effect of job strain on allostatic load (β = −0.02, 95% CI −0.10 to 0.06), which could indicate a more confounded measure of job strain.

The results of this study indicate that social and material adversity in early life may predispose individuals to a higher sensitivity to psychosocial stressors, which in themselves are more common among the materially deprived. Further research is warranted to study if this interaction between early life adversity and later psychosocial exposures can be generalised, and whether interventions to decrease adversity in early life can prevent harmful stress reactions in adulthood.

In conclusion, exposure to an adverse social environment in adolescence was associated with increased vulnerability to job strain in mid-life, indicating that sensitivity to stress and social inequalities in health may both be partially determined by material factors in early life.

## Supporting Information

Figure S1 **The distribution of the Job Strain and Allostatic Load indices.**
(TIF)
