# Supplementary figures and images for: Social Adversity in Adolescence Increases the Physiological Vulnerability to Job Strain in Adulthood: A Prospective Population-Based Study
Source: PLoS One. 2012 Apr 25;7(4):e35967. doi: 10.1371/journal.pone.0035967 (PMC3338487; doi:10.1371/journal.pone.0035967)

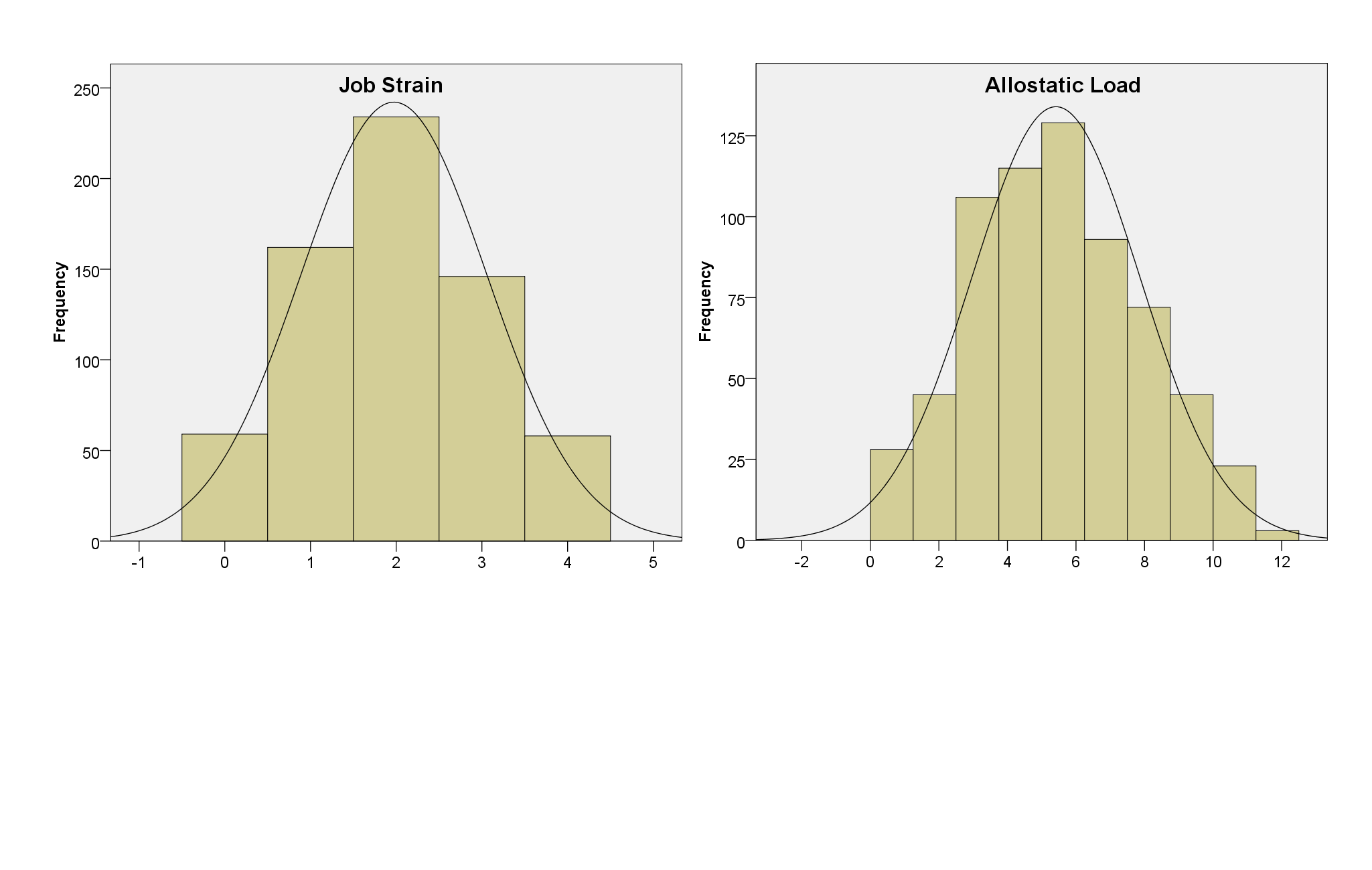

Supplement: Figure S1 — The distribution of the Job Strain and Allostatic Load indices. (TIF) [file pone.0035967.s001.tif]
